# Screening for diabetes mellitus among tuberculosis patients in Southern Nigeria: a multi-centre implementation study under programme settings

**DOI:** 10.1038/srep44205

**Published:** 2017-03-10

**Authors:** Ngozi Ekeke, Kingsley N. Ukwaja, Joseph N. Chukwu, Charles C. Nwafor, Anthony O. Meka, Eruke E. Egbagbe, Festus O. Soyinka, Isaac Alobu, Ifeanyi Agujiobi, Samuel Akingbesote, Osagie Igbinigie, Job B. Offor, Nelson O. Madichie, Chukwuka Alphonsus, Moses C. Anyim, Obinna K. Mbah, Daniel C. Oshi

**Affiliations:** 1 Medical Department, German Leprosy and TB Relief Association, Enugu, Enugu State, Nigeria; 2 Department of Medicine, Federal Teaching Hospital, Abakaliki, Ebonyi State, Nigeria; 3 Department of Medicine, University of Benin Teaching Hospital, Benin City, Edo State, Nigeria; 4 Ogun State Tuberculosis and Leprosy Control Programme, Ministry of Health, Abeokuta, Ogun State, Nigeria; 5 Ebonyi State Tuberculosis and Leprosy Control Programme, Ministry of Health, Abakaliki, Ebonyi State, Nigeria; 6 Enugu State Tuberculosis and Leprosy Control Programme, Ministry of Health, Enugu, Enugu State, Nigeria; 7 Ondo State Tuberculosis and Leprosy Control Programme, Ministry of Health, Akure, Ondo State, Nigeria; 8 Edo State Tuberculosis and Leprosy Control Programme, Ministry of Health, Benin City, Edo State, Nigeria; 9 Cross River State Tuberculosis and Leprosy Control Programme, Ministry of Health, Calabar, Cross River State, Nigeria; 10 Department of Community Health and Psychiatry, University of West Indies, Jamaica

## Abstract

Implementation studies are recommended to assess the feasibility and effectiveness of programmes. In Nigeria, little is known about the burden of diabetes mellitus (DM) among tuberculosis (TB) patients. The objective of this study was to determine screening efficacy, prevalence of DM and determinants of DM among TB patients. We report on a multi-centre implementation study carried-out in 13 health facilities in six States of Southern Nigeria. All newly diagnosed TB patients registered from March to October 2015 were screened for DM using current World Health Organisation guidelines. Overall, 2094 TB patients were evaluated, 196 (9.4%) were found to have DM. The prevalence of newly diagnosed DM was 5.5% (115/2094). DM prevalence varied according to age group; occurring in 2.2% of patients aged ≤ 25 years and 16.9% in patients aged (56–65) years. The additional yield of DM was 59% while the number needed to screen to detect a new case of DM was 18. Factors associated with DM were; age >40 years (aOR2.8, CI 2.1–3.9), rural residence (aOR2.3, 1.6–3.2), private health facility care (aOR2.0, 1.4–2.7), and having an occupation that engages in vigorous activity (aOR0.6, 0.4–0.9). The burden of DM among TB patients is high. Prioritization of DM screening for TB patients is indicated.

Containing the burden of tuberculosis (TB) and Diabetes mellitus (DM) remains a global health challenge. In 2015, it was estimated that 10.4 million incident cases of TB occurred globally, while 1.4 million persons died from it^1^. In the same year, the corresponding estimated prevalence and mortality due to DM were 415 million and 5 million, respectively^2^. If current trends continue, it is projected that by 2040, 642 million persons will be affected by DM^2^, 80% of whom will be residing in low- and middle-income countries where TB burden is highest^2^. DM is an important risk factor for developing TB. Systematic reviews have shown that the overall risk of TB in persons with DM is three times higher than in the general population^3^,^4^,^5^. In addition, persons with dual TB-DM disease have worse TB treatment outcomes with increased delays to sputum culture conversion, higher risk of relapse, death and drug resistance^5^,^6^,^7^. Conversely, TB and its treatment can impair glycaemic control – impacting the clinical management of DM^5^,^8^.

In recognition of the burden of DM and TB, the World Health Organization (WHO) and the International Union against Tuberculosis and Lung Disease (The Union) launched the Collaborative Framework for Care and Control of Tuberculosis and Diabetes to guide policy makers and programme managers in combating the TB-DM epidemic^9^. However, recent studies have shown that DM prevalence among TB cases is variable. It ranges from about 50% reported by some studies in India, about 30% in Mexico, less than 10% in the United States, to even lower values in certain African countries, Spain and China^10^,^11^,^12^,^13^,^14^,^15^,^16^. As reported levels of co-morbidity vary highly, National TB Control Programmes (NTPs) will need to decide whether such DM screening is needed. Where necessary, the most cost-effective approach for each setting, for example targeted screening or screening of all patients should be determined^17^.

Nigeria ranks third among 30 countries with estimated highest burden of TB in the world as well as third among African countries with highest burden of DM^1^,^2^. Considering that two thirds of DM patients in Africa are not aware that they have the disease, screening for DM among TB patients may contribute to early diagnosis^2^. In Nigeria, some research has been conducted on the prevalence of DM among TB patients^18^,^19^. However, as these studies were limited to hospitals located in a single urban area, it is unlikely to reflect the disease burden in rural areas or other geographical and cultural settings in the country. As WHO/The Union have called for operational and clinical research to build up and strengthen local evidence base for action in combating the dual epidemic^17^, quality data to guide this process in Nigeria is crucial but lacking. The aim of this study was to investigate the epidemiology of DM among TB patients in Nigeria. The specific objectives were; to determine 1) the proportion of TB patients with DM, 2) the additional yield of newly diagnosed DM cases, 3) the number needed to screen (NNS) to find a new case of DM, and 4) the determinants of DM among TB patients in Southern Nigeria.

## Results

### General characteristics

A total 2132 new and previously-treated TB patients (aged ≥ 15 years) were registered for treatment during the study period. However, 38 (1.8%) who were previously-treated TB patients were excluded from the study because they were not eligible. All eligible 2094 newly diagnosed TB patients completed the screening. The socio-demographic characteristics of the patients are shown in Table 1. The mean age was 40.7 years (standard deviation [SD] ±15.7). There were more males (56.4%) than females (43.6%), and majority resided in urban areas (51.9%). Furthermore, 95.3% of the patients had pulmonary tuberculosis (PTB), and 62.8% received care at a public facility. Among patients with PTB, 533 (26.7%) had smear-negative TB while 1463 (73.3%) had smear-positive/bacteriologically-confirmed TB. History of current smoking was elicited in 4.8% while 19.5% of patients had TB/HIV co-infection. In terms of level of physical activity according to occupation, 49.8% of patients had occupations that were largely sedentary, 32.9% had moderate activity and 17.3% had occupation associated with vigorous activity.

### Prevalence of diabetes mellitus

The overall prevalence of DM among TB patients studied stratified by age, sex, residence, type of TB, physical activity and other factors, are as shown in Table 2. Of the 2094 patients, 196 (9.4%, 95% CI 8.2–10.7) were found to have DM. Of these, 81 (3.9%) were previously known to have DM, while 115 (5.5%) were newly diagnosed. The prevalence of DM was 2.2% (95% CI 1.1–4.4) among those aged ≤ 25 years compared with 15.1% (95% CI 11.5–19.5) and 16.9% (95% CI 12.6–22.3) among those aged (46–55) and (56–65) years, respectively (P < 0.001). The prevalence of DM did not differ according to type of TB, smoking status, HIV co-infection or gender. However, the prevalence of DM was 13.3% (95% CI 11.4–15.5) among rural residents compared to 5.7% (95% CI 4.5–7.2) among urban residents (P < 0.001). Similarly, the prevalence of DM among TB patients who received care at a public facility was 6.6% (95% CI 5.4–8.1) compared to 14.0% (95% CI 11.8–16.6) among patients who received care at a private facility (P < 0.001). The prevalence of DM among patients with sedentary occupation was 10.2% (95% CI 8.5–12.2). This was slightly higher in those engaged in occupation involving moderate/vigorous physical activity, but the difference was not statistically significant (p = 0.3).

### Additional yield of diabetes mellitus and number needed to screen (NNS)

Of the 2094 TB patients, 196 (9.4%) were found to have DM, of whom 115 (5.5%) were newly-diagnosed cases. The additional yield of DM cases on screening was 59%. The NNS to detect one new case of DM was 18. Among patients aged 25 years or less, the NNS was 119, and among those aged (56–65) years the NNS was 9 (Table 3). The additional yield of males was similar to that of females (60% versus 60%). The NNS to diagnose one male DM patient was 18 compared to 19 for females. The additional yield of screening patients resident in rural areas for DM was 60% versus 58% for patients in urban areas. However, the NNS to diagnose one DM case among patients resident in the rural area was approximately 13 compared to 30 for urban residents. Regarding HIV status, additional yield among HIV-negative DM patients was 60.7% versus 48.5% for HIV-positive DM patients. However, the NNS to diagnose one HIV-negative DM patient was 17 compared to 26 for HIV-positive DM patients.

### Risk factors associated with DM

The results of multivariable logistic regression analysis are presented in Table 4. After adjusting for potential confounders, risk factors such as age >40 years (aOR 2.8, CI 2.1–3.9), rural residence (aOR 2.3, 1.6–3.2), and private facility care (aOR 2.0, 1.4–2.7), were found to be independently associated with increased risk of DM among TB patients. However, having an occupation that engages in vigorous activity (aOR 0.6, 0.4–0.9) was found to be independently associated with decreased risk of DM among TB patients.

## Discussion

This study found a DM prevalence of 9.4% among newly diagnosed TB patients. The additional yield of DM was 59% while the NNS to detect a new case of DM was 18. The prevalence of DM among patients who were older than 40 years was far higher than among younger persons; sex on the other hand was not associated with the occurrence of DM. The additional yield of DM was greater among older patients, persons who sought care at a public facility, rural residents and HIV-negative persons. In all, factors favouring occurrence of DM among TB patients in the study group were older age (over 40 years), private facility care and rural residence. Conversely, patients engaged in occupations with vigorous activity were less likely have DM.

The DM prevalence of 9.4% among newly diagnosed TB patients found in this study is much higher than was observed in a pilot study in a Nigerian city as well as in the general population, but was lower than a prevalence of 12.3% observed in some health facilities in the same city^2^,^18^,^19^. Our finding was also comparable with the results of studies carried-out in Ethiopia (8.3%), Uganda (8.5%) and USA (11.4%)^11^,^12^,^20^. However, DM prevalence among TB patients reported in this study was higher than the rates reported from China (6.3%), Spain (5.9%), Guinea-Bissau (2.8%) and Benin (1.9%); but lower than the rates reported from Taiwan (29.5%), Southern-Mexico (29.3%) and India (25.3%)^13^,^14^,^15^,^16^,^21^,^22^,^23^. Reason/s for the differences in prevalence might be due to disparities in patient background and disease burden. In Guinea-Bissau and Benin where low DM prevalence was observed, the burden of TB in both countries is low^21^,^22^. In addition, the low DM prevalence observed in these settings may also be explained by the high burden of TB among younger persons, who in general have a lower incidence of DM than those aged (40 years or over)^24^. Furthermore, differences in dietary habits and the screening methods used for DM diagnosis might contribute to the variation in prevalence of DM among TB patients in various regions^13^,^14^,^15^,^16^,^21^,^22^,^23^.

DM screening was conducted in large public and private facilities across Southern Nigeria under programme conditions with minimal additional training to the health facility workers. This suggests that the active screening strategy of TB patients for DM is feasible and acceptable. This finding may indicate the possibility of DM screening for TB patients at primary health care (PHC) facilities, and the need to establish its referral linkages in order to ensure quality DM services to patients diagnosed with DM. This would however require the establishment of a functional referral system to ensure access to quality DM services at appropriate secondary and tertiary health facilities.

The NNS to detect a new case of DM among TB patients was 18. This number is higher than the findings in India which showed large variations in NNS to detect DM among TB patients^16^. These differences may be due to the high prevalence of DM in India, the quality of screening test used and the countrywide standard procedure applied for DM screening in India^25^. In addition, we found that NNS needed to detect one case of DM among TB patients decreases as the age increases. Owing to the differences between the various studies, it is crucial to understand the relationship between TB and DM in different regions around the world in order to establish appropriate priorities and adapt the needs of National TB Control Programmes. In high TB burden countries with weak public health systems, the current recommendation is to rule out TB among diabetics and vice versa^15^,^17^,^26^. Given the low NNS noted in our setting to detect a DM case, and the need to sustain DM-TB screening programmes in Nigeria, the most cost-effective approach would be to prioritise older TB patients for targeted screening.

The strength of this study is that we were able to implement this screening programme in a routine programme setting across multiple regions and facilities with minimal additional costs and training. The study has a few limitations, however. First, it was carried out at high-end performing TB centres, therefore, the results may not be representative of all TB treatment settings e.g. PHC settings in Southern Nigeria. Secondly, we utilised FBG for diagnosis; FBG testing has low sensitivity and may fail to detect some persons with DM. However, FBG has been recommended as the initial DM screening test in resource-limited settings^5^. The HbA1c has also been recently recommended for the diagnosis of DM among TB patients; however, it is limited by being expensive, cumbersome to perform and inappropriate for screening individuals within routine general health services^17^. Third, we excluded previously-treated TB patients in this study. Although it could be argued that previously-treated TB patients are a risk group for the development of co-morbid TB-DM, we excluded them because in some of the facilities where the survey was carried-out, these patients were still being investigated for drug-resistant TB. Also, because the optimal time for DM screening among TB patients is not clear^3^, we excluded previously-treated TB patients because it will be difficult to ascertain if the DM diagnosed in this group is as a result of persistent TB disease or due to previous exposures to TB medications both of these situations has been shown to cause persistent hyperglycemia^3^,^27^,^28^,^29^. Besides, we wanted to establish the burden of DM in treatment-naive TB patients receiving first-line anti-TB treatment in order to provide evidence to advocate for systematic DM screening among the largest burden of adult TB patients in Nigeria. Finally, we did not undertake anthropometric measurements (e.g., body mass index, waist circumference, etc.) of the respondents. A recent study in Pakistan found anthropometric heterogeneity among TB patients with/without DM – those with known or newly-diagnosed DM being more likely to be obese compared with those without DM^30^. Thus, such anthropometric measurements need to be considered in future studies in our setting.

This study has potential major policy implications. First, DM screening among TB patients is important for early diagnosis of DM – especially in Nigeria where two thirds of DM cases may be unaware of their status. The current screening strategy can improve early diagnosis of DM and facilitate necessary interventions. Second, it is especially important to screen older TB patients (≥40 years). Third, the study highlights the need for similar studies in PHC settings to estimate the burden of DM and the feasibility of decentralizing DM-TB services in rural and remote under-resourced settings.

## Conclusions

The burden of DM among TB patients in Nigeria is high. Screening can detect more than half of undiagnosed DM among newly diagnosed TB patients. In this and similar under-resourced settings where prioritization for DM screening is necessary, screening TB patients who are aged >40 years may yield the greatest benefits. Furthermore, screening among TB patients residing in rural areas and those engaged in occupations with sedentary and moderate physical activity is expected to increase the additional yield of DM cases.

## Materials

### Study design

This was a cross-sectional implementation study conducted under programme conditions in six States in Southern Nigeria. The study was conducted over an eight-month period from March to October 2015.

### Setting and study population

Nigeria is divided into two regions (north and south), and six geo-political zones (three per region) with each having between five to seven States. The study was conducted in the three zones in Southern Nigeria. Patients were recruited through a two-stage sampling technique. First, two States were selected from each of the three geo-political zones in southern Nigeria through simple random sampling. The study states include Edo and Cross River (south-south zone), Enugu and Ebonyi (south-east zone), and Ogun and Ondo (south-west zone). In each selected State, the top-two (three in Ondo State) health facilities notifying and treating the highest proportion of TB patients were identified and selected for the survey. In all, 13 health facilities which offer both TB and diabetes services were selected for the survey.

### Eligibility

The inclusion criteria were all adults (≥15 years) with newly-diagnosed TB starting treatment. Patients were ineligible if they had previously-treated TB.

### Screening procedure

All newly-diagnosed adult TB patients aged (≥15 years) attending the study health facilities were interviewed on their first visit for commencement of TB treatment. DM status was assessed using patients self-reported history regarding DM. All eligible patients irrespective of known diagnosis of DM were investigated as follows: Random blood glucose (RBG) test was performed – if the level was less than 110 mg/dl, no further action was taken. If the RBG was ≥110 mg/dl, then the patient was asked to return for fasting blood glucose (FBG) test the next day. A glucometer (ACCU-CHEK Active Glucose Monitoring System, Roche Diabetes Care Limited, Burgess Hill, UK) was used for screening DM. Standard WHO diagnostic criteria were used for making a diagnosis of diabetes mellitus, i.e., fasting blood glucose (FBG) value of ≥126 mg/dl^19^. Patients who were found to be diabetic were referred to the diabetes clinic of their facility for further evaluation and management. Moreover, known DM patients were educated on the need to continue follow-up care for DM.

A standardized proforma was used to collect patients’ socio-demographic and clinical variables from the TB registers as well as the screening results^17^. Physical activity was assessed based on patient’s occupation and classified as ‘sedentary’, ‘moderate’ and ‘vigorous’ according to WHO classification^31^. Diagnosis of TB was made in line with the WHO/NTP guidelines^32^. Patients found positive for *Mycobacterium tuberculosis* on smear microscopy or based on MTB RIF Xpert test were considered to have bacteriological-positive TB. Smear-negative TB was defined by negative smear (or Xpert) results with radiology, clinical and/or histology findings consistent with active TB, followed by a clinicians’ decision to treat with a full course of anti-TB chemotherapy. Extra-pulmonary TB (EPTB) was based on histological or strong clinical evidence consistent with active EPTB, and a clinicians’ decision to treat with a full course of TB chemotherapy. HIV counseling and testing was provided to all persons with presumed TB during submission of sputum specimen for microscopy.

### Statistical analysis

Data were double-entered, validated for consistency and analysed using Epi Info. *Dataset available as a supplementary information* (SI). Patients’ demographic and clinical characteristics were described in terms of frequencies and percentages. The main outcomes for analysis were the number and proportion of TB patients with known and/or newly diagnosed DM, further stratified by age, sex, type of TB, residence and physical activity. Bivariate analysis was performed to assess the association of these factors with DM prevalence. Odds ratios (ORs) with 95% confidence intervals (CIs) were used to describe associations between groups. Multivariable logistic regression analysis was performed to estimate adjusted ORs using the full model fits. NNS was calculated for all patients whose DM status was assessed. Additional yield of new cases of DM was calculated using the following formula: (newly diagnosed DM cases × 100)/(known DM cases + newly diagnosed DM cases).

### Ethics approval

An informed verbal consent was obtained from each participant before enrolment in the study. We confirm that all blood sugar testing were performed in accordance with relevant WHO guidelines and regulations in use in clinical practice in Nigeria. The Ethics and Research Advisory Board of German Leprosy and TB Relief Association, Nigeria approved the study. Approval was also obtained from the State TB Control Programme in six states selected for the project.

## Additional Information

**How to cite this article:** Ekeke, N. *et al*. Screening for diabetes mellitus among tuberculosis patients in Southern Nigeria: a multi-centre implementation study under programme settings. *Sci. Rep.* **7**, 44205; doi: 10.1038/srep44205 (2017).

**Publisher's note:** Springer Nature remains neutral with regard to jurisdictional claims in published maps and institutional affiliations.

## Supplementary Material

Supplementary Dataset 1

## Figures and Tables

**Table 1:** Characteristics of tuberculosis patients attending the study facilities (n = 2094).

Characteristics	n (%)
Age (years)	
≤40	1190 (56.8)
>40	904 (43.2)
Sex	
Female	913 (43.6)
Male	1181 (56.4)
Residence	
Rural	1007 (48.1)
Urban	1087 (51.9)
Type of TB	
EPTB	98 (4.7)
PTB	1996 (95.3)
Facility type	
Private	778 (37.2)
Public	1316 (62.8)
HIV status	
Negative	1686 (80.5)
Positive	408 (19.5)
Current smoker	
No	101 (4.8)
Yes	1993 (95.2)
Physical activity	
Sedentary	1042 (49.8)
Moderate	689 (32.9)
Vigorous	363 (17.3)

TB: tuberculosis, HIV: human immunodeficiency virus, EPTB: extrapulmonary TB, PTB: pulmonary TB.

**Table 2:** Prevalence of diabetes among TB patients in Southern Nigeria 2015 (n = 2094).

	Total TB patients evaluated for DM	TB patients with DM	Prevalence of DM	P -value
	N	n	% (95%CI)	
Total	2094	196	9.4 (8.2–10.7)	
Age (years)				<0.001
≤25	358	8	2.2 (1.1–4.4)	
26–35	585	44	7.5 (5.7–9.9)	
36–45	458	41	9.0 (6.7–11.9)	
46–55	305	46	15.1 (11.5–19.5)	
56–65	231	39	16.9 (12.6–22.3)	
>65	157	18	11.5 (7.4–17.4)	
Sex				0.93
Female	913	86	9.4 (7.7–11.5)	
Male	1181	110	9.3 (7.8–11.1)	
Residence				<0.001
Rural	1007	134	13.3 (11.4–15.5)	
Urban	1087	62	5.7 (4.5–7.2)	
Type of TB				0.26
EPTB	98	6	6.1 (2.8–12.7)	
PTB	1996	190	9.5 (8.3–10.9)	
Facility type				<0.001
Private	778	109	14.0 (11.8–16.6)	
Public	1316	87	6.6 (5.4–8.1)	
HIV status				0.33
Negative	1686	163	9.7 (8.3–11.2)	
Positive	408	33	8.1 (5.8–11.1)	
Current Smoker				0.87
No	1993	187	9.4 (8.2–10.7)	
Yes	101	9	8.9 (4.8–16.1)	
Physical activity				0.30
Sedentary	1042	106	10.2 (8.5–12.2)	
Moderate	689	55	8.0 (6.2–10.3)	
Vigorous	363	35	9.6 (7.0–13.1)	

TB: tuberculosis, HIV: human immunodeficiency virus, EPTB: extrapulmonary TB, PTB: pulmonary TB.

**Table 3:** Additional yield of new cases of DM and NNS to diagnose a new case of DM among TB patients in Southern Nigeria 2015 (n = 2094).

Variables	TB patients	Number of patients	Number of newly	Additional yield	NNS
	evaluated for DM	with previous DM	diagnosed DM	[c/(b + c) × 100]	
	[a]	[b]	[c]	%	[a/c]
Total	2094	81	115	58.7	18.2
Age (years)					
≤25	358	5	3	37.5	119.3
26–35	585	17	27	61.4	21.7
36–45	458	19	22	53.7	20.8
46–55	305	18	28	60.9	10.9
56–65	231	14	25	64.1	9.2
>65	157	8	10	55.6	15.7
Sex					
Female	913	37	49	60.0	18.6
Male	1181	44	66	60.0	17.9
Residence					
Rural	1007	55	79	60.0	12.8
Urban	1087	26	36	58.1	30.2
Type of TB					
EPTB	98	3	3	50.0	32.7
PTB	1996	78	112	60.0	17.8
Facility type					
Private	778	54	55	50.5	14.1
Public	1316	27	60	70.0	21.9
HIV status					
Negative	1686	64	99	60.7	17.0
Positive	408	17	16	48.5	25.5
Current Smoker					
No	1993	77	110	58.8	18.1
Yes	101	4	5	55.6	20.2
Physical activity					
Sedentary	1042	41	34	45.3	30.7
Moderate	689	21	65	75.6	10.6
Vigorous	363	19	16	45.7	22.7

TB: tuberculosis, HIV: human immunodeficiency virus, EPTB: extrapulmonary TB, PTB: pulmonary TB. DM: diabetes mellitus, NNS: number needed to screen.

**Table 4:** Factors associated with DM among TB patients attending the study facilities (n = 2094).

Variables	OR (95% CI)	aOR (95% CI)	Adjusted P -value
Age (years)			
≤40	1	1	<0.001
>40	2.5 (1.9–3.4)	2.8 (2.1 3.9)	
Sex			
Female	1.0 (0.8–1.4)	1.1 (0.8–1.6)	0.46
Male	1	1	
Residence			
Rural	2.5 (1.9–3.5)	2.3 (1.6–3.2)	<0.001
Urban	1	1	
Type of TB			
EPTB	1	1	
PTB	1.6 (0.7–3.7)	1.5 (0.6–3.5)	0.36
Facility type			
Private	2.3 (1.7–3.1)	2.0 (1.4–2.7)	<0.001
Public	1	1	
HIV status			
Negative	1.2 (9.8–1.8)	1.3 (0.9–2.0)	0.18
Positive	1	1	
Current Smoker			
No	1.1 (0.5–2.1)	1.4 (0.7–3.0)	0.36
Yes	1	1	
Physical activity			
Sedentary	1.3 (0.9–1.8)	1.2 (0.8–1.7)	0.32
Moderate	1	1	
Vigorous	1.2 (0.8–1.9)	0.6 (0.4–0.9)	0.027

TB: tuberculosis, HIV: human immunodeficiency virus, EPTB: extrapulmonary TB, PTB: pulmonary TB.

## References

[b1] World Health Organization. Global tuberculosis report 2016. Geneva: World Health Organization; 2016 [cited 2016 Nov. 1]. http://www.who.int/tb/publications/global_report/en/.

[b2] International Diabetes Federation. *IDF Diabetes, 7 ed.* Brussels, Belgium: International Diabetes Federation http://www.diabetesatlas.org (2015).

[b3] Dooley K. E. & Chaisson R. E. Tuberculosis and diabetes mellitus: convergence of two epidemics. Lancet Infect. Dis. 9, 737–746 (2009). 19926034 10.1016/S1473-3099(09)70282-8 PMC2945809

[b4] Ruslami R., Aarnoutse R. E., Alisjahbana B., van der Ven A. J. & van Crevel R. Implications of the global increase of diabetes for tuberculosis control. Trop. Med. Int. Health 15, 1289–1299 (2010). 20955495 10.1111/j.1365-3156.2010.02625.x

[b5] Pizzol D. . Tuberculosis and diabetes: current state and future perspectives. Trop. Med. Int. Health. 21, 694–702 (2016). 27102229 10.1111/tmi.12704

[b6] Baker M. A. . The impact of diabetes on tuberculosis treatment outcomes: a systematic review. BMC. Med. 9, 81 (2011). 21722362 10.1186/1741-7015-9-81 PMC3155828

[b7] Mi F. . Diabetes mellitus and tuberculosis: pattern of tuberculosis, two-month smear conversion and treatment outcomes in Guangzhou, China. Trop. Med. Int. Health 18, 1379–1385 (2013). 24112411 10.1111/tmi.12198

[b8] Van-Cromphaut S., Vanhorebeek I. & Van-den-Berghe G. Glucose metabolism and insulin resistance in sepsis. Curr. Pharm. Des. 14, 1887–1899 (2008). 18691100 10.2174/138161208784980563

[b9] World Health Organization and the International Union Against Tuberculosis and Lung Disease. Collaborative Framework for Care and Control of Tuberculosis and Diabetes. WHO/HTM/TB/2011.15. Geneva, Switzerland: WHO, 2011. 26290931

[b10] Viswanathan V. . Prevalence of diabetes and pre-diabetes and associated risk factors among tuberculosis patients in India. PLOS. ONE 7, e41367 (2012). 22848473 10.1371/journal.pone.0041367 PMC3406054

[b11] Magee M. J. . Diabetes mellitus and risk of all-cause mortality among patients with tuberculosis in the state of Georgia, 2009–2012. Ann. Epidemiol. 24, 369–375 (2014). 24613196 10.1016/j.annepidem.2014.01.012 PMC4011933

[b12] Kibirige D., Ssekitoleko R., Mutebi E. & Worodria W. Overt diabetes mellitus among newly diagnosed Ugandan tuberculosis patients: a cross sectional study. BMC. Infect. Dis. 13, 122–129 (2013). 23497232 10.1186/1471-2334-13-122 PMC3599954

[b13] Jiménez-Corona M. E. . Association of diabetes and tuberculosis: impact on treatment and post-treatment outcomes. Thorax. 68, 214–220 (2013). 23250998 10.1136/thoraxjnl-2012-201756 PMC3585483

[b14] Wang Q. . Prevalence of type 2 diabetes among newly detected pulmonary tuberculosis patients in China: a community based cohort study. PLOS. One. 18, e82660 (2013). 10.1371/journal.pone.0082660 PMC3867381 24367535

[b15] Moreno-Martínez A. . Factors associated with diabetes mellitus among adults with tuberculosis in a large European city, 2000–2013. Int. J. Tuberc. Lung Dis. 19, 1507–1512 (2015). 26614193 10.5588/ijtld.15.0102

[b16] Shidam U. G., Roy G., Sahu S. K., Kumar S. V. & Ananthanarayanan P. H. Screening for diabetes among presumptive tuberculosis patients at a tertiary care centre in Pondicherry, India. Int. J. Tuberc. Lung Dis. 19, 1163–1168 (2015). 26459527 10.5588/ijtld.14.0971

[b17] Harries A. D. . Diabetes mellitus and tuberculosis: programmatic management issues. Int. J. Tuberc. Lung Dis. 19, 879–886 (2015). 26162352 10.5588/ijtld.15.0069 PMC4497633

[b18] Olayinka A. O., Anthonia O. & Yetunde K. Prevalence of diabetes mellitus in persons with tuberculosis in a tertiary health centre in Lagos, Nigeria. Indian J. Endocrinol. Metab. 17, 486–489 (2013). 23869307 10.4103/2230-8210.111646 PMC3712381

[b19] Ogbera A. O. . Undiagnosed diabetes mellitus in tuberculosis: A Lagos report. Indian J. Endocrinol. Metab. 18, 475–479 (2014). 25143901 10.4103/2230-8210.137488 PMC4138900

[b20] Workneh M. H., Bjune G. A. & Yimer S. A. Prevalence and associated factors of diabetes mellitus among tuberculosis patients in South-Eastern Amhara Region, Ethiopia: a cross sectional study. PLoS. One. 11, e0147621 (2016). 26808967 10.1371/journal.pone.0147621 PMC4726615

[b21] Ade S. . Low prevalence of diabetes mellitus in patients with tuberculosis in Cotonou, Benin. Public Health Action. 5, 147–9 (2015) 26400387 10.5588/pha.14.0110 PMC4487487

[b22] Haraldsdottir T. L. . Diabetes mellitus prevalence in tuberculosis patients and the background population in Guinea-Bissau: a disease burden study from the capital Bissau. Trans. R. Soc. Trop. Med. Hyg. 109, 400–407 (2015) 25918218 10.1093/trstmh/trv030

[b23] Chang J. T. . Effect of type-2 diabetes mellitus on the clinical severity and treatment outcome in patients with pulmonary tuberculosis: a potential role in the emergency of multi drug resistance. J. Formos. Med. Assoc. 110, 372–381 (2011). 21741005 10.1016/S0929-6646(11)60055-7

[b24] World Health Organization, Benin Ministry of Health. Final report of the STEPS survey in Benin. Geneva, Switzerland: WHO, 2007. http://www.who.int/chp/steps/2007_STEPS_Report_Benin.pdf Accessed January 2017.

[b25] Members of tuberculosis-diabetes study group. Screening of patients with tuberculosis for diabetes mellitus in India. Trop. Med. Int. Health. 18, 636–645 (2013). 23458555 10.1111/tmi.12084

[b26] World Health Organization. Screening for type 2 diabetes. Report of a World Health Organization and International Diabetes Federation Meeting, 2003. WHO/NMH/MNC/03.1. Geneva, Switzerland: WHO, 2003. http://www.who.int/diabetes/publications/en/screening_mnc03.pdf Accessed March 2016.

[b27] Baghaei P., Marjani M., Javanmard P., Tabarsi P. & Masjedi M. R. Diabetes mellitus and tuberculosis facts and controversies. J. Diabetes Metab. Disord. 12, 58 (2013). 24360398 10.1186/2251-6581-12-58 PMC7962555

[b28] Zack M. B., Fulkerson L. L. & Stein E. Glucose intolerance in pulmonary tuberculosis. Am. Rev. Respir. Dis. 108, 1164–1169 (1973). 4746573 10.1164/arrd.1973.108.5.1164

[b29] Atkin S., Masson E., Bodmer C., Walker B. & White M. Increased insulin requirement in a patient with type 1 diabetes on rifampicin. Diab. Med. 10, 202 (1992). 10.1111/j.1464-5491.1993.tb00086.x 8508626

[b30] Aftab H. . High prevalence of diabetes and anthropometric heterogeneity among tuberculosis patients in Pakistan. Trop. Med. Int. Health. (*in* press), doi: 10.1111/tmi.12842. 28102021

[b31] Joint FAO/WHO/UNU. Human Energy Requirements. Report of a Joint FAO/WHO/UNU Expert Consultation, Rome, 17–24 Oct. 2001. Rome, FAO/WHO/UNU, 2004.

[b32] National Tuberculosis and Leprosy Control Programme, Revised Workers’ Manual, Sixth ed., Federal Ministry of Health, Abuja, Nigeria, 2015.

